# PAK2 promotes proliferation, migration, and invasion of lung squamous cell carcinoma through the LIMK1/cofilin signaling pathway

**DOI:** 10.7555/JBR.37.20230317

**Published:** 2024-06-03

**Authors:** Congcong Wang, Junyan Wang, Ruifeng Xu, Qiushuang Li, Xia Huang, Chenxi Zhang, Baiyin Yuan

**Affiliations:** 1 College of Life and Health Sciences, Wuhan University of Science and Technology, Wuhan, Hubei 430081, China; 2 Central Laboratory, Nanjing Chest Hospital, Affiliated Nanjing Brain Hospital of Nanjing Medical University, Nanjing, Jiangsu 210029, China

**Keywords:** PAK2, lung squamous cell carcinoma, LIMK1, cofilin

## Abstract

Although p21-activated kinase 2 (PAK2) is an essential serine/threonine protein kinase, its role in the progression of lung squamous cell carcinoma (LUSC) has yet to be fully understood. We analyzed *PAK2* mRNA levels, DNA copy numbers, and protein levels by quantitative reverse transcription-PCR and immunohistochemical staining in both human LUSC tissues and adjacent normal tissues. Then, we performed colony formation assays, cell counting kit-8 assays, Matrigel invasion assays, wound healing assays, and xenograft models in nude mice to investigate the functions of PAK2 in LUSC progression. We demonstrated that *PAK2* mRNA levels, DNA copy numbers, and protein levels were upregulated in human LUSC tissues, compared with adjacent normal tissues. Additionally, higher PAK2 expression was associated with poorer prognosis in LUSC patients. In the *in vitro* study, we found that PAK2 promoted cell growth, migration, invasion, epithelial-mesenchymal transition, and cell morphology regulation in LUSC cells. Mechanistically, PAK2 promoted tumor cell proliferation, migration, and invasion by regulating actin dynamics through the LIMK1/cofilin signaling pathway. Our findings indicate that the PAK2/LIMK1/cofilin signaling pathway may serve as a potential clinical marker and therapeutic target for LUSC.

## Introduction

Lung cancer is one of the most common cancers worldwide^[1]^. Studies have reported that approximately 85%–90% of lung cancer cases are non-small cell lung cancer (NSCLC)^[2]^, with two main subtypes: lung squamous cell carcinoma (LUSC) and lung adenocarcinoma (LUAD)^[3]^. Unfortunately, most NSCLC patients are diagnosed at an advanced metastatic stage, resulting in a high mortality rate and a nearly 16% five-year survival rate^[4]^. Therefore, clarifying the mechanisms contributing to cancer development and progression is crucial for improving early diagnosis and treatment for NSCLC patients.

The p21-activated kinases (PAKs) are serine/threonine kinases that are classified into two groups, PAK1–3 and PAK4–6, both displaying an N-terminal GTPase-binding domain and a C-terminal kinase domain^[5–6]^. PAKs are indispensable for multiple cellular processes, including cell movement, cell attachment in the cellular environment, angiogenesis, tumor invasion, tumor migration, and the regulation of cell cycle and mitosis^[7]^. One study reported that the expression and activation levels of PAK1 were higher in LUSC cells than in LUAD cells, which were associated with increased migration and invasion capabilities in LUSC cells^[8]^. Furthermore, inhibiting PAK3 has been shown to potentially suppress the metastasis of invasive lung cancer^[9]^.

PAK2, another member of group Ⅰ PAKs, is broadly expressed *in vivo* and is involved in the progression of various cancer types^[10]^. For instance, the expressions of PAK2 and Ser20-phosphorylated PAK2 (pSer20PAK2) were upregulated, and their overexpressions were associated with poor pathology and medical prognosis in gastric cancer^[11]^. Additionally, PAK2 promoted cell proliferation, and its expression was correlated with metastasis and mortality of breast cancer^[12]^. The p-PAK2 Ser^20^ expression was upregulated in ovarian cancer cells, and p-PAK2 Ser^20^ promoted cell migration and invasion but did not affect cell proliferation and apoptosis^[13]^. Furthermore, PAK2 was reported to be consistently overexpressed in melanoma and prostate adenocarcinoma cells^[14–15]^. However, the effect of PAK2 on LUSC progression has yet to be further analyzed, because its underlying molecular mechanisms are intricate and unclear.

PAKs are the activated downstream effectors of the RAC family small GTPase 1 (RAC1) and cell division cycle 42 (CDC42) signaling pathways, and they regulate actin cytoskeleton dynamics by phosphorylating the LIM domain kinase 1 (LIMK1)^[16]^, a serine/threonine kinase that is phosphorylated at threonine 508 by PAK1, PAK4, or PAK6^[16–17]^. Cofilin is the predominant factor that promotes actin filament (F-actin) severing and depolymerization^[18]^, whereas the activated LIMK1 phosphorylates the downstream target cofilin at serine 3 (Ser-3) and suppresses its actin depolymerization activity^[19]^.

In the present study, we aimed to investigate the association between PAK2 expression in human LUSC tissues and survival of LUSC patients, and further unravel the role and underlying mechanisms of PAK2 in LUSC cancer progression, as a promising prognostic molecular marker and a possible therapeutic target for LUSC.

## Materials and methods

### Patients and tissue samples

NSCLC and the matched adjacent normal tissue samples, including 24 LUAD cases and 30 LUSC cases, were collected from patients who underwent surgery without chemotherapy or radiotherapy at the Nanjing Chest Hospital between October 2016 and September 2021. Characteristics of the patients with NSCLC are shown in ***Supplementary Table 1*** (available online). After resection, NSCLC and the matched adjacent normal tissues were immediately snap-frozen in liquid nitrogen and stored at −80 ℃ until used for quantitative reverse transcription-PCR (qRT-PCR) to examine *PAK2* mRNA levels and DNA copy numbers. The present study was conducted following the Declaration of Helsinki, and the protocol was approved by the Institutional Review Board of Nanjing Chest Hospital. The informed consent was obtained from each patient. All procedures performed in the present study involving human participants were in line with the ethical standards of institutions and national research committees.

### Cell culture and transfection

LUSC cell lines (H226 and H520) were obtained from the Cell Bank, Chinese Academy of Sciences (Shanghai, China). Cells were cultured in an RPMI 1640 medium (Gibco, GrandIsland, NY, USA) supplemented with 10% activated fetal bovine serum (FBS; VivaCell, Shanghai, China) and 1% streptomycin and penicillin (MeilunBio, Dalian, China). The pLKO.1-TRC lentiviral vector was used to generate the *PAK2* knockdown plasmid. The control shRNA (shCTL) sequence for GFP was 5ʹ-GCAAGCTGACCCTGAATTCAT-3ʹ. The shRNA sequences for *PAK2* knockdown were 5ʹ-CCGGCGGGATTTCTTAAATCGATGTCTCGAGACATCGATTTAAGAAATCCCGTTTTTTG-3ʹ (sh*PAK2*-1), and 5ʹ-CCGGCCATCCATGTTGGCTTTGATGCTCGAGCATCAAAGCCAACATGGATGGTTTTTTG-3ʹ (sh*PAK2*-3). The siRNA (Genepharma, Shanghai, China) sequences targeting *PAK2* were 5ʹ-GGAGGUUGCUAUCAAACAAAU-3ʹ (si*PAK2*-1) and 5ʹ-GACUAAGAUGACAGAUGAAGA-3ʹ (si*PAK2*-2). The control siRNA sequence was 5ʹ-UUCUCCGAACGUGUCACGUTT-3ʹ.

RNAs (2 μg) were transfected into H226 and H520 cells using Lipofectamine 3000 (Cat. #L3000150, Invitrogen, Carlsbad, CA, USA). After 48 h, the cells were harvested and analyzed. *PAK2* was amplified with primers (forward 5ʹ-GCTGGATATCTGCAGAATTCATGTCTGATAACGGAGAACTGG-3ʹ; reverse 5ʹ-GTACCGAGCTCGGATCCACGGTTACTCTTCATTGCTTC-3ʹ) and incorporated into the pCDNA3.1 vector to create pcDNA-*PAK2* plasmid. Similarly, *LIMK1* was amplified with primers (forward 5ʹ-GCTGGATATCTGCAGAATTCATGAGGTTGACGCTACTTTGTTG-3ʹ; reverse 5ʹ-GTACCGAGCTCGGATCCGTCGGGGACCTCAGGGTG-3ʹ) and incorporated into the pCDNA3.1 vector to create pCDNA-*LIMK1* plasmid. *LIMK1* was amplified with primers (forward 5ʹ-GCTGGATATCTGCAGAATTCATGAGGTTGACGCTACTTTGTTG-3ʹ; reverse 5ʹ-TGGTACCGAGCTCGGATCCTCACAGATCCTCTTCTGAGATGAGTTTTTGTTCGTCGGGGACCTCAGGGTG-3ʹ) and incorporated into the pCDNA3.1 vector to create pCDNA-*LIMK1*-Myc plasmid.

### Immunohistochemical (IHC) staining and scoring of PAK2 in tissue microarrays

Tissue microarrays (Cat. #HluS180Su01-M-164, Shanghai Outdo Biotech, Shanghai, China) containing 60 paired LUSC and adjacent normal tissues were used to determine the PAK2 expression. ***Supplementary Table 2*** (available online) displays the characteristics of patients whose samples were used for the tissue microarray analysis. Heat-mediated antigen retrieval was performed by using a citrate buffer at pH 6.0, and tissue sections were incubated with primary antibodies (rabbit monoclonal anti-human PAK2, Cat. #ab76293, Abcam, Cambridge, UK) at 4 ℃ overnight. The UltraSensitive SP (rabbit) IHC kit (Cat. #KIT-9706, Maixin Biology, Fuzhou, China) was used for signal detection, and hematoxylin was employed as a counterstain.

Subsequently, sections were observed under a light microscope, and the images were captured. The expression level of PAK2 was calculated based on the IHC mean staining intensity (ranging from 0 to 130). The mean staining intensity was classified as follows: < 60 = 0; 61–70 = 1; 71–80 = 2; and 81–130 = 3. The positive cell percentage was determined by calculating the proportion of cytoplasm positive-staining cells and was scored as follows: positive cells < 25% (0 points), positive cells 26%–50% (1 point), positive cells 51%–75% (2 points), and positive cells 76%–100% (3 points). The score of PAK2 expression (the staining index) was obtained by multiplying the mean staining intensity and the positivity score. The samples were classified into low (0–2) and high expression (3–9) groups based on the staining index^[11]^.

Sections were scanned using the StrataFAXSII system (TissueGnostics, Vienna, Austria), and the images of digital tumor areas were quantitatively analyzed using StrateQuest analysis software (version 7.0.1.165, TissueGnostics) to evaluate the PAK2 expression considering prognosis and clinicopathologic features. ***Table 1*** displays the correlations between the expression levels of PAK2 and clinicopathological factors in LUSC patients.

**Table 1:** Correlations between the PAK2 protein expression and clinicopathological characteristics in LUSC patients

Characteristics	Total (*n*)	PAK2 expression		*χ* ^2^	*P*-value
		Low (*n*)	High (*n*)		
Age (years)				0	0.833
≥60	29	8	21		
<60	29	13	16		
Sex				0	0
Male	60	25	35		
Female	0	0	0		
p-TNM stages				5.19	0.023
Ⅰ	13	9	4		
Ⅱ–Ⅲ	47	16	31		
Tumor depth (pT)				0.23	0.631
T1–T2	42	16	26		
T3–T4	13	9	4		
Lymph node metastasis (pN)				1.15	0.283
N0–N1	49	22	27		
N2	5	1	4		
Missing data because of incomplete provision of patient information.					

### qRT-PCR

Total RNA was extracted from tissues and cells using TRIzol reagent (Cat. #15596-018, Life Technologies, Carlsbad, CA, USA), and the RNA quality was assessed using the NanoDrop 300 spectrophotometer (ThermoFisher Scientific, Waltham, MA, USA) following the manufacturer's instructions. Subsequently, 1 μg of total RNA from each sample was reverse-transcribed to cDNA with a reverse transcription kit (Cat. #R222-01, Vazyme, Nanjing, China), and AceQ Universal SYBR qPCR Master Mix (Cat. #Q511-02, Vazyme) was used to perform qRT-PCR on the BIO-RAD CFX96 instrument (BIO-RAD, Hercules, CA, USA). All primers were designed with Primer Premier 5.0 as follows: *PAK2* forward 5ʹ-TGAGCACACCATCCATGTTGG-3ʹ and reverse 5'-AGGTCTGTAGTAATCGAGCCC-3ʹ; *GAPDH* forward 5ʹ-GGAGCGAGATCTCTCCAAAAT-3ʹ and reverse 5ʹ-GGCTGTTGTCATACTTCTCATGG-3'; cyclin A2 (*CCNA2*) forward 5'-CATTGGTCCCTCTTGATT-3' and reverse 5ʹ-TAACCTCCATTTCCCTAAG-3ʹ; cyclin D1 (*CCND1*) forward 5ʹ-ATTTCCAATCCGCCCTCC-3ʹ and reverse 5ʹ-GGCTTCGATCTGCTCCTGG-3ʹ; cyclin E 1 (*CCNE1*) forward 5ʹ-CAGGGAGACCTTTTACTTG-3ʹ and reverse 5ʹ- CCATCTGTCACATACGCA-3ʹ; *P21* forward 5ʹ-CCTGGCACCTCACCTGCTCT-3ʹ and reverse 5ʹ-CGGCGTTTGGAGTGGTAGAA-3ʹ; and *P27* forward 5ʹ-CGGCTAACTCTGAGGACAC-3ʹ and reverse 5ʹ-CTGTTCTGTTGGCTCTTTTGT-3ʹ. The relative mRNA level was normalized with that of *GAPDH*. The ΔΔCt value was calculated following the manufacturer's protocol.

### Western blotting assay

Western blotting assay was performed following standard protocols. Briefly, total cell proteins were extracted from LUSC cells using RIPA buffer (Cat. #P0013B; Beyotime, Shanghai, China), and the protein concentration was measured using a BCA Protein Assay kit (Cat. #P0012, Beyotime). Equal protein amounts (20 μg) were separated on SDS-PAGE gels and transferred to PVDF membranes. After blocking with 5% non-fat milk, membranes were incubated with primary antibodies at 4 ℃ overnight, and then with horseradish peroxidase-conjugated secondary antibodies at room temperature on the next day^[4]^. The antibodies targeting the following proteins were used: GAPDH (1∶3000, Cat. #AP0063, Bioworld Technology, Nanjing, China), β-actin (1∶3000, Cat. #sc-47778, Santa Cruz Biotechnology, Dallas, Texas), PAK2 (1∶1000, Cat. #76293, Abcam, Cambridge, MA, USA), cofilin (1∶1000, Cat. #BS2183, Bioworld Technology), p-cofilin (Ser-3; 1∶1000, Cat. #3311S, Cell Signaling Technology, Danvers, MA, USA), LIMK1 (1∶1000, Cat. #A16664, ABclonal, Wuhan, China), p-LIMK1 (1∶1000, T508; Cat. #AP0387, ABclonal), N-cadherin (1∶1000, Cat. #13116, Cell Signaling Technology), vimentin (1∶1000, Cat. #ab92547, Abcam), E-cadherin (1∶1000, Cat. #BS1098, Bioworld Technology), and Myc-Tag (1∶1000, Cat. #AE009, ABclonal).

### Cell counting kit-8 (CCK8) assay

After transfection, cells (1 × 10^3^ cells/well) were seeded into a 96-well plate in triplicate. Cells were incubated for 1, 2, 3, and 4 days. Then the cells were supplemented with 100 μL of CCK8 solution (Cat. #A311-01, Vazyme), and further incubated at 37 ℃ for 2 h. The absorbance was detected at 450 nm with a microtiter plate reader.

### Matrigel invasion assay

Costar transwell chambers with an 8-μm aperture (Cat. #3422, Corning Costar, NY, USA) were used for the invasion assay. Cells from different treatments (1 × 10^5^) were suspended in a 100 μL RPMI 1640 medium without FBS, and added to each upper transwell chamber. Then, 800 μL RPMI 1640 medium containing 10% FBS was added to the lower chambers. After incubating for 24 and 48 h, Matrigel and cells remaining in the upper chambers were removed with cotton swabs. Cells that invaded the lower surface of the chamber membrane were fixed with 4% formaldehyde and stained with crystal violet.

### Clonal formation assay

Cells from different treatments (1 × 10^3^) were plated into 6-well plates and cultured in RPMI 1640 medium containing 10% FBS for eight days. Next, cells were washed with PBS, fixed with 4% paraformaldehyde for 10 min, and stained with crystal violet. Digital images were captured for subsequent analyses.

### Wound healing assay

The treated cells were inoculated into 6-well plates and starved in a medium containing 1% FBS for 12 h. After 90%–100% cell convergence, the cell layer was scratched off with a sterilized 200 μL pipette tip. Images at multiple scratch points (0, 24, 48, and 72 h) after scratching were captured with a microscope (Olympus) at 10× magnification.

### Xenograft models in nude mice

Four-week-old male BALB/c nude mice (GemPharmatech, Nanjing, China), housed under specific pathogen-free conditions, were used for tumorigenesis assays. H226 cells that stably expressed low PAK2 levels were constructed through lentivirus-mediated shRNA interference of *PAK2* (sh*PAK2*). Then, H226 cells (2 × 10^6^) were subcutaneously injected into the flanks of nude mice (*n* = 7 for each group). The tumor size was measured once a week, and the volume was calculated by 0.44 × length (mm) × width (mm)^2^. Eleven weeks later, mice were euthanized by CO_2_ asphyxiation, and tumors were excised. The study was approved by the Institutional Animal Care and Use Committee (IACUC) of Wuhan University of Science and Technology. All protocols in the present study were executed strictly following national and international laws, including the Guide for the Care and Use of Laboratory Animals.

### Immunofluorescence staining

Cells from different treatments were seeded on rat tail type Ⅰ collagen-coated glass coverslips in 12-well plates. After culture, these cells were fixed with 4% paraformaldehyde for 20 min and permeabilized with 0.2% Triton X-100 (KRbio, Jinan, China) at room temperature for five minutes. Cells were incubated with tetramethylrhodamine-labeled phalloidin (Cat. #p1951, Sigma-Aldrich, St. Louis, MO, USA) for F-actin filament staining and 4',6-diamidino-2-phenylindole (DAPI; Cat. #D6584, Sangon Biotech, Shanghai, China) for nuclear staining. The cell cross-sectional surface area, determined as a perimeter along the cellular surface, and the cell length and width, determined as the length along the maximum and minimum axes, were measured using ImageJ (National Institutes of Health, http://rsb.info.nih.gov/ij). The expression level of F-actin was quantified by measuring the grayscale value of phalloidin staining per area in cells.

### Statistical analysis

Statistical analysis was performed using GraphPad Prism version 5.0 software (GraphPad Software, USA). For two-group comparisons, statistical analyses were conducted by two-tailed unpaired Student's *t*-test, and the data were presented as mean ± standard deviation. All experiments were repeated at least three times. Pearson's Chi-squared test was used to analyze the correlations between PAK2 expression levels and clinicopathological parameters in lung cancer patients. Kaplan-Meier survival curves were generated using Prism software, and the statistical significance of the intergroup differences in data was evaluated using the log-rank test. *P* < 0.05 was considered statistically significant.

## Results

### Upregulated PAK2 transcription levels and copy numbers in LUSC tissues

**Figure 1:**
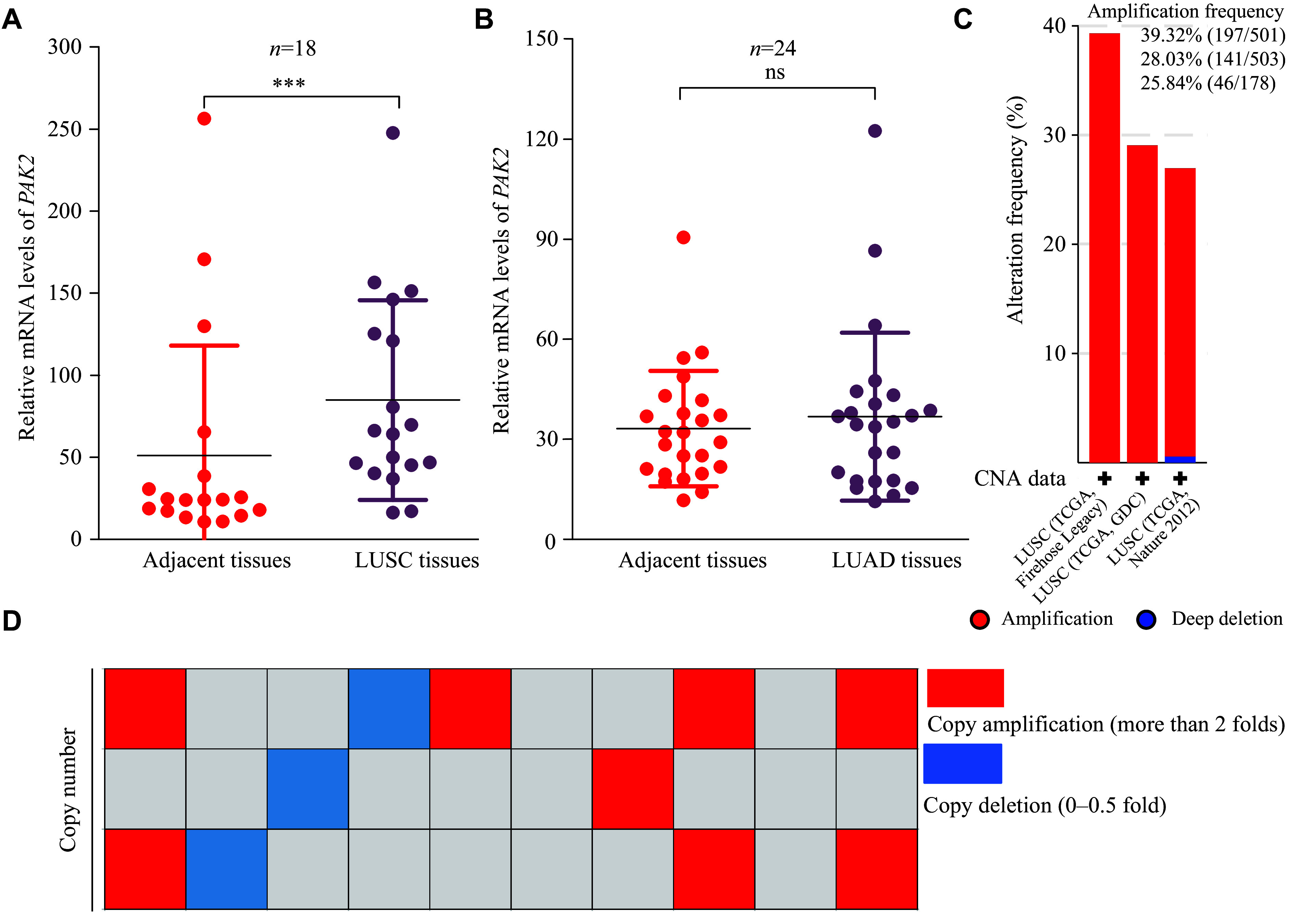
The upregulated *PAK2* transcription levels and amplified copy numbers in LUSC tissues. A: The expression levels of *PAK2* in human LUSC and paired adjacent normal tissues by quantitative reverse transcription-PCR (qRT-PCR) analysis (*n* = 18). B: The expression levels of *PAK2* in human LUAD and paired adjacent non-tumor tissues by qRT-PCR analysis (*n* = 24). C: *PAK2* CNV analysis in LUSC using the cBioPortal online database (http://cbioportal.org). Amplification frequencies of the three groups were also shown. D: qRT-PCR validation of *PAK2* copy number alterations in LUSC and paired adjacent normal tissue samples (*n* = 30). Red indicates samples with amplified copy numbers, and blue indicates samples with deleted copy numbers. Data are presented as mean ± standard deviation. Statistical significance was assessed using a two-tailed unpaired Student's *t*-test. ^***^P < 0.001. Abbreviations: ns, not significant; LUSC, lung squamous cell carcinoma; LUAD, lung adenocarcinoma; CNV, copy number variation.

We detected *PAK2* mRNA levels in human LUSC and the paired adjacent normal lung tissues to explore the effect of PAK2 on LUSC progression. The results showed that *PAK2* mRNA levels were significantly higher in LUSC tissues than in adjacent normal tissues (***Fig. 1A***), but no significant differences in *PAK2* mRNA levels were detected between LUAD and adjacent normal tissues (***Fig. 1B***). Similar results were also obtained from online The Cancer Genome Atlas (TCGA) and Clinical Proteomic Tumor Analysis Consortium (CPTAC) databases (***Supplementary Fig. 1***, available online).

Subsequently, we investigated the genomic DNA copy number alterations of *PAK2* using the online database cBioPortal (http://cbioportal.org), and found a significant amplification of *PAK2* copy number in LUSC tissues (***Fig. 1C***). We further examined the genomic DNA copy numbers of *PAK2* in 30 LUSC tissues and the paired adjacent normal tissues through relative quantification, incorporating the standard curve method using a qRT-PCR assay, to verify the results obtained from the online database cBioPortal. The results showed that copy numbers of *PAK2* were considerably amplified in human LUSC tissues, and eight LUSC tissues with high copy numbers displayed high *PAK2* mRNA expression levels (***Fig. 1D***), suggesting that PAK2 may participate in LUSC progression.

### A high PAK2 expression level was correlated with a poor prognosis in LUSC patients

**Figure 2:**
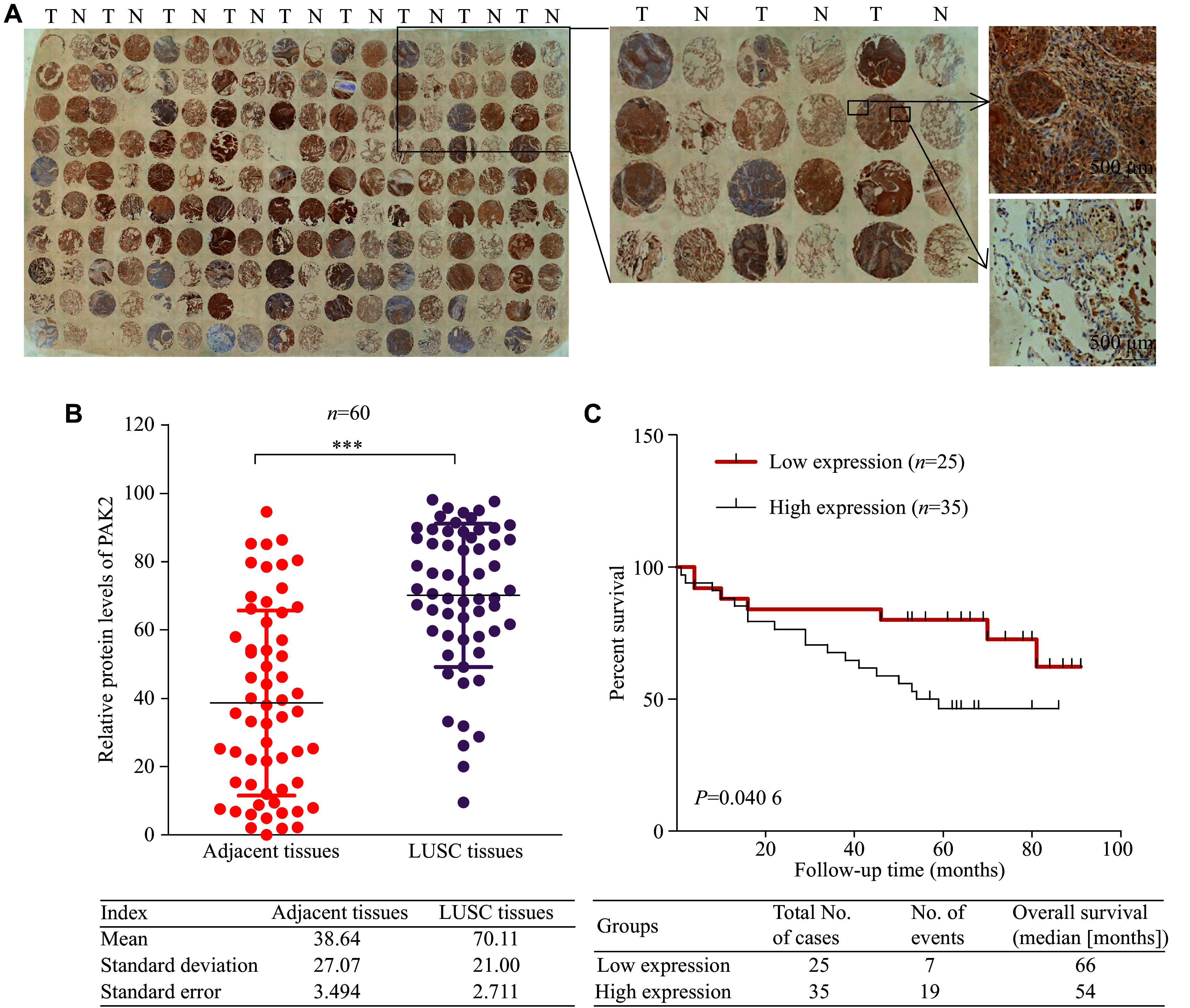
A high PAK2 expression level was correlated with a poor prognosis in LUSC patients. A: Expression levels of PAK2 protein in TMAs (90 human LUSC and paired adjacent normal tissues) were determined through immunohistochemical staining. Enlarged boxes indicate PAK2 staining examples in LUSC and non-tumor tissues. T for tumor, N for non-tumor. On the right are the magnified images. Scale bar, 500 μm. B: Protein levels of PAK2 in LUSC and paired adjacent non-tumor tissues (*n* = 60). C: Kaplan-Meier survival analysis based on staining index scores. Patients were divided into two groups with high (*n* = 35) or low (*n* = 25) levels of PAK2. Data are presented as mean ± standard deviation. Statistical analyses were performed by two-tailed unpaired Student's *t*-test for two-group comparisons. ^***^P < 0.001. Abbreviations: TAMs, tissue microarrays; LUSC, lung squamous cell carcinoma.

We performed the IHC staining assays to detect expression levels of the PAK2 protein in 90 human LUSC tissues and the paired adjacent normal tissues. The data from 60 usable samples indicated that expression levels of the PAK2 protein were significantly higher in LUSC tissues than in adjacent non-tumor tissues (***Fig. 2A*** and ***2B***). Next, we analyzed the correlations between PAK2 expression levels and LUSC clinicopathological characteristics. The samples were classified into high and low PAK2 groups based on PAK2 expression score (the staining index). The results showed that high expression levels of the PAK2 protein were significantly correlated with advanced tumor stages (***Table 1***). Additionally, we performed survival analysis to evaluate the association between PAK2 expression levels and patient survival, and found that patients with high PAK2 expression levels had worse survivals than those with low expression levels (***Fig. 2C***), indicating that PAK2 may play an oncogenic role in the LUSC progression.

### Silencing the PAK2 expression inhibited the proliferation, migration, and invasion of LUSC cells

**Figure 3:**
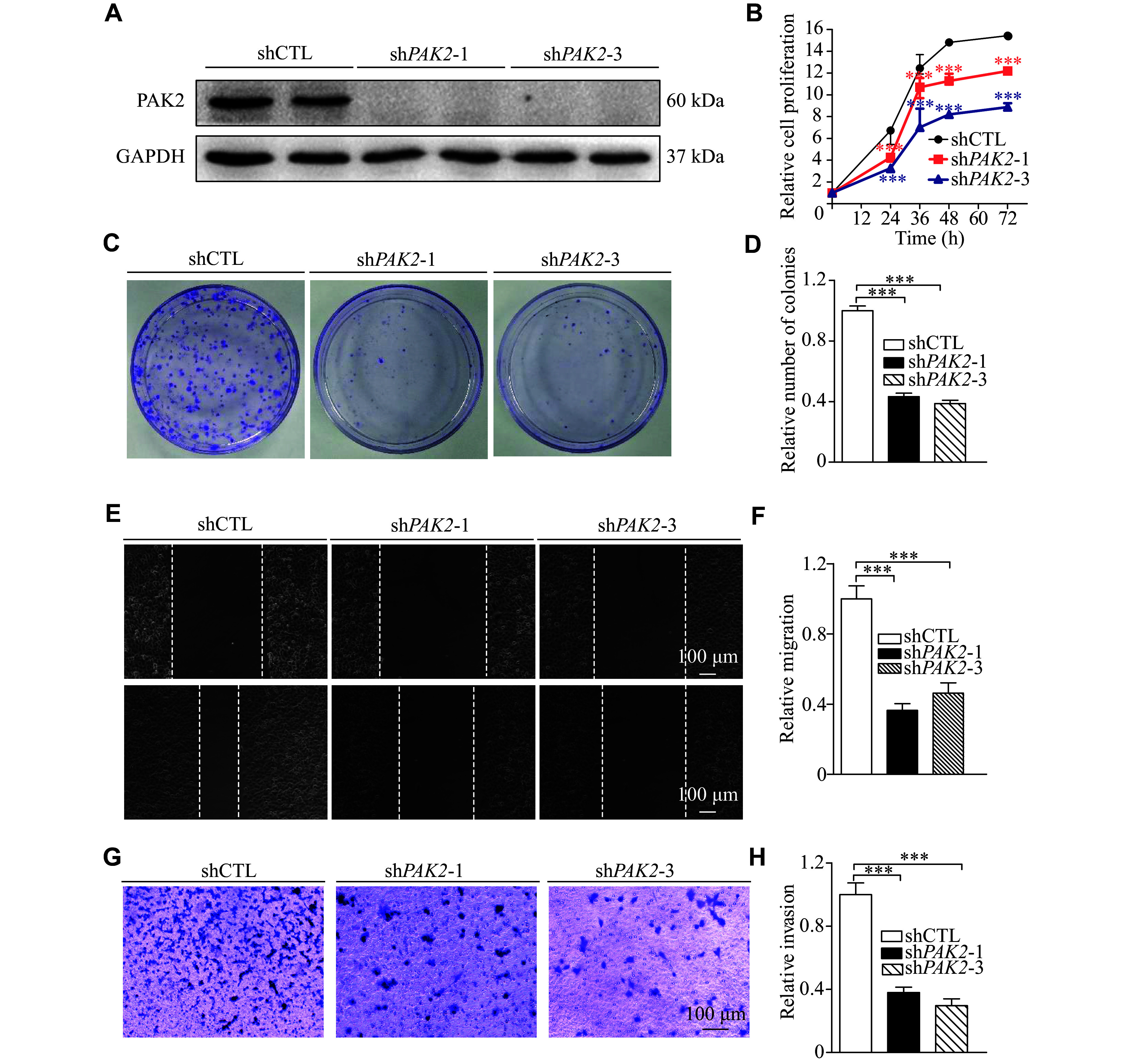
Silencing the PAK2 expression suppressed LUSC cell proliferation, migration, and invasion. A: The stable *PAK2* knockdown (mediated by sh*PAK2*-1 and sh*PAK2*-3) and control (shCTL) H226 cells were generated, and Western blotting analysis was performed to determine the *PAK2* knockdown efficacy. GAPDH served as the loading control. B: The CCK8 assay was used to detect the proliferation of sh*PAK2*-1, sh*PAK2*-3, and control H226 cells. C and D: The colony formation growth assay was used to test the cell colony formation ability. Images show the shCTL, sh*PAK2*-1, and sh*PAK2*-3 H226 cell colonies grown from a single cell after a culture of eight days (C). GraphPad software was used to count the number of shCTL, sh*PAK2*-1, and sh*PAK2*-3 H226 cell colonies (D). E and F: Wound healing assay and quantification analysis in *PAK2* knockdown H226 cells. Scale bar, 100 μm. G and H: Matrigel invasion assay and quantification analysis in *PAK2* knockdown H226 cells. Scale bar, 100 μm. Data are presented as mean ± standard deviation. Statistical analyses were performed by two-tailed unpaired Student's *t*-test for two-group comparisons. ^***^P < 0.001, compared with the shTCL group.

The expression levels of *PAK2* in three LUSC cell lines are shown in ***Supplementary Fig. 2*** (available online). The H226 cell line was used to analyze the biological function of PAK2 in LUSC cells. Lentiviruses carrying shRNA sequences were transfected into H226 cells to generate stable *PAK2* knockdown cells (sh*PAK2*-1 and sh*PAK2*-3). The *PAK2* knockdown efficiency was assessed by Western blotting. Our results showed that expression levels of PAK2 were significantly decreased in sh*PAK2*-1 and sh*PAK2*-3 transfected cells, compared with the control cells (shCTL; ***Fig. 3A***).

Subsequently, we performed CCK8 and colony formation assays, wound healing assays, Matrigel invasion assays, and TUNEL staining to investigate the effects of PAK2 on proliferation, colony formation, migration, invasion, and apoptosis of H226 cells, respectively. The results showed that the silencing of PAK2 expression in H226 cells inhibited proliferative activity (***Fig. 3B***) and colony formation (***Fig. 3C*** and ***3D***), suppressed the wound closure (***Fig. 3E*** and ***3F***), and reduced cell invasive activity (***Fig. 3G*** and ***3H***), but did not affect the cell apoptosis (***Supplementary Fig. 3***, available online). Similar results regarding the effects of PAK2 on cell proliferation, migration, and invasion were also observed in H520 cells (***Supplementary Fig. 4***, available online).

In contrast to the results of *PAK2* knockdown in the aforementioned experiments, cells overexpressing PAK2, generated by transfecting plasmids that overexpress *PAK2*, exhibited significantly increased proliferation, migration, and invasion (***Supplementary Fig. 5***, available online).

### PAK2 promoted tumor growth and epithelial-mesenchymal transition (EMT) *in vivo*

**Figure 4:**
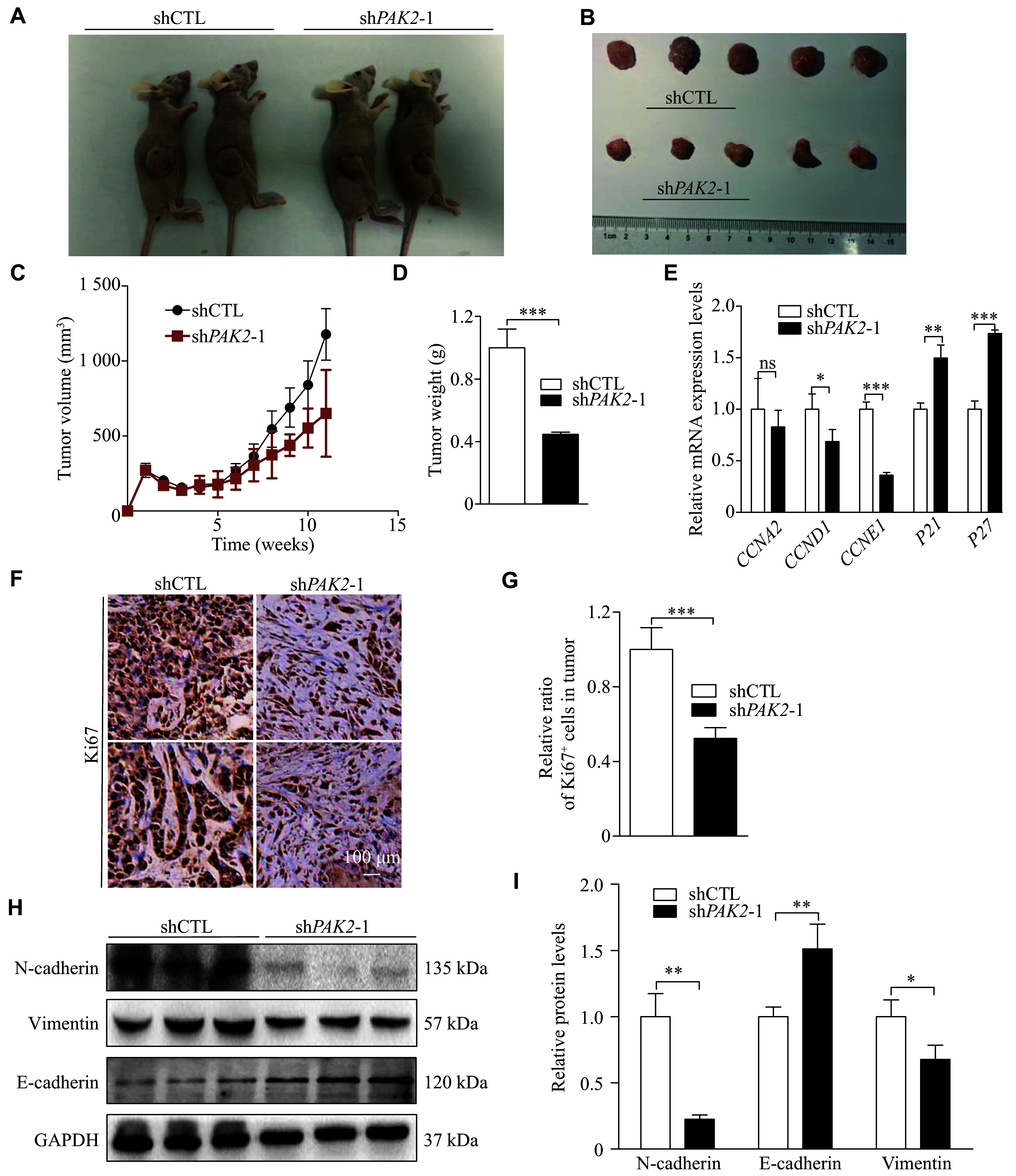
PAK2 promoted tumor growth and induced epithelial-mesenchymal transition (EMT) *in vivo*. A: The *in vivo* tumor formation of shCTL and sh*PAK2* H226 cells (*n* = 2; some data not shown). All the xenograft tumors of each group were collected at the end time point (11 weeks). B: Comparison of the subcutaneously xenografted tumors of sh*PAK2* H226 cells and control cells in nude mice. Top: shCTL (*n* = 5); bottom: sh*PAK2*-1 (*n* = 5). C: Xenograft tumor growth of control cells and *PAK2* knockdown H226 cells in mice. Tumor volume was monitored every seven days post-inoculation (*n* = 5). D: Tumor weight statistics for the ShCTL and sh*PAK2* groups (*n* = 5). E: Quantitative reverse transcription-PCR analysis was performed to detect the mRNA expression levels of cyclin A2 (C*CNA2*), cyclin D1 (*CCND1*), cyclin E1 (*CCNE1*), *P21*, and *P27* in xenograft tumors. F and G: Ki67 staining (F) of the resected tumors and quantification analysis (G). Scale bar, 100 μm. H and I: EMT markers, including E-cadherin, N-cadherin, and vimentin expression levels were detected through Western blotting in H226 cells (H). The Image J software was used to measure the grayscale values of target bands (I). Data are presented as mean ± standard deviation. Statistical analyses were performed by two-tailed unpaired Student's *t*-test for two-group comparisons. ^*^P < 0.05, ^**^P < 0.01 and ^***^P < 0.001.

To further determine the biological function of PAK2 in LUSC cell proliferation *in vivo*, H226 cells with stable shCTL or sh*PAK2* were subcutaneously injected into immunodeficient nude mice, and the tumor volume was measured once a week. Xenograft tumors were localized at the injection site in immunodeficient mice and were harvested at 70 days post-injection (***Fig. 4A*** and ***4B***). Consistent with the *in vitro* results, the xenograft tumors derived from *PAK2*-deficient H226 cells displayed a significantly decreased growth rate, compared with the shCTL group (***Fig. 4C*** and ***4D***).

Subsequently, we assessed the effects of PAK2 on cell cycle progression to identify how PAK2 promotes LUSC cell proliferation. qRT-PCR results showed that low PAK2 expression significantly reduced the expression levels of cyclin D and cyclin E, but increased the expression levels of *P21* and *P27* (***Fig. 4E***). Similarly, IHC staining of Ki67 revealed that PAK2 promoted LUSC cell proliferation *in vivo* (***Fig. 4F*** and ***4G***). Western blotting results also showed that *PAK2* knockdown significantly upregulated the expression level of E-cadherin, an epithelial marker, but inhibited the expression levels of mesenchymal markers, such as N-cadherin and vimentin, in H226 cells. These results indicated that *PAK2* knockdown suppressed the EMT process (***Fig. 4H*** and ***4I***), suggesting that PAK2 may regulate tumor growth and the EMT process *in vivo*.

### *PAK2* knockdown repressed the LIMK1/cofilin signaling

**Figure 5:**
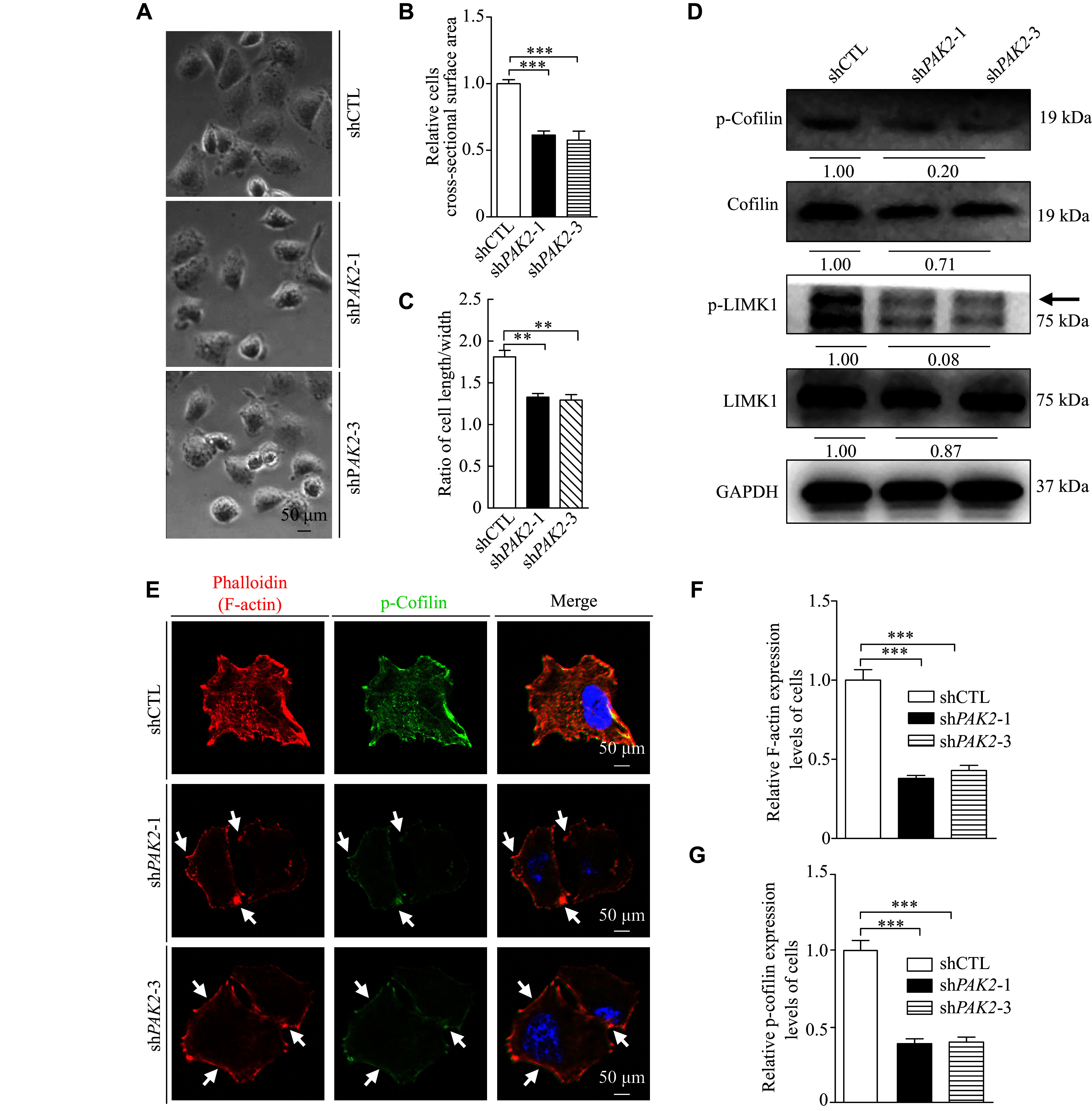
*PAK2* knockdown suppressed the LIMK1/cofilin signaling. A–C: Bright-field images of shCTL, sh*PAK2*-1, and sh*PAK2*-3 H226 cells. Scale bar, 50 μm. D: Western blotting was used to detect p-LIMK1, LIMK1, p-cofilin, and cofilin levels in H226 cells. GAPDH was used as a control. E–G: Immunofluorescence staining of F-actin and p-cofilin in H226 cells with *PAK2* silencing. DAPI (blue), F-actin (red), p-cofilin (green), and merged images are shown. Arrows indicate the enriched F-actin and co-localized p-cofilin at the cell peripheries. Scale bar, 50 μm. Quantification of F-actin (F) and p-cofilin (G) expression levels in shCTL, sh*PAK2*-1, and sh*PAK2*-3 H226 cells. Data are presented as mean ± standard deviation. Statistical analyses were performed by two-tailed unpaired Student's *t*-test for two-group comparisons. ^**^P < 0.01 and ^***^P < 0.001.

Compared with shCTL cells, an obvious morphological change was observed in sh*PAK2* cells. Specifically, most sh*PAK2* cells were noticeably smaller and rounder, based on cell cross-sectional surface area and cell length/width ratios (***Fig. 5A***–***5C***).

It has been reported that PAKs regulate LIMK1/cofilin cascade activities, a crucial signal transduction pathway in tumor metastasis^[15,20]^. In addition, LIMK1 regulates tumor cell migration and invasion by modulating cofilin activity to influence actin cytoskeleton remodeling^[21–23]^. Thus, we examined the levels of LIMK1, p-LIMK1, cofilin, and p-cofilin in sh*PAK2* and shCTL cells. p-LIMK1 and p-cofilin levels were significantly decreased, cofilin levels were mildly decreased, and LIMK1 levels were not significantly changed in sh*PAK2* cells, compared with shCTL cells (***Fig. 5D***). We also detected the levels of LIMK1, p-LIMK1, cofilin, and p-cofilin in xenograft tumors, and corroborated the results of Western blotting performed in cells (***Supplementary Fig. 6***, available online). These results indicate that *PAK2* knockdown may inhibit the LIMK1/cofilin signaling pathway.

Cofilin is necessary for actin dynamics, assisting in cell motility, cytokinesis, and cellular morphology regulation. Therefore, phalloidin staining was performed to detect the F-actin levels. Compared with shCTL cells, F-actin levels were significantly decreased in sh*PAK2* cells and notably enriched at the cell peripheries (***Fig. 5E*** and ***5F***), suggesting that *PAK2* knockdown inhibited actin assembly. In addition, the immunostaining results showed that F-actin and p-cofilin were co-located in both shCTL and sh*PAK2* cells. Compared with shCTL cells, p-cofilin levels in sh*PAK2* cells were significantly decreased, which was consistent with the reduced F-actin levels. Moreover, p-cofilin exhibited a strong staining distribution pattern and co-localization with F-actin at the cell edge (***Fig. 5E*** and ***5G***).

### LIMK1 overexpression in PAK2 deficient cells partially rescued the impaired proliferative, migratory, and invasive capacities of the cells

**Figure 6:**
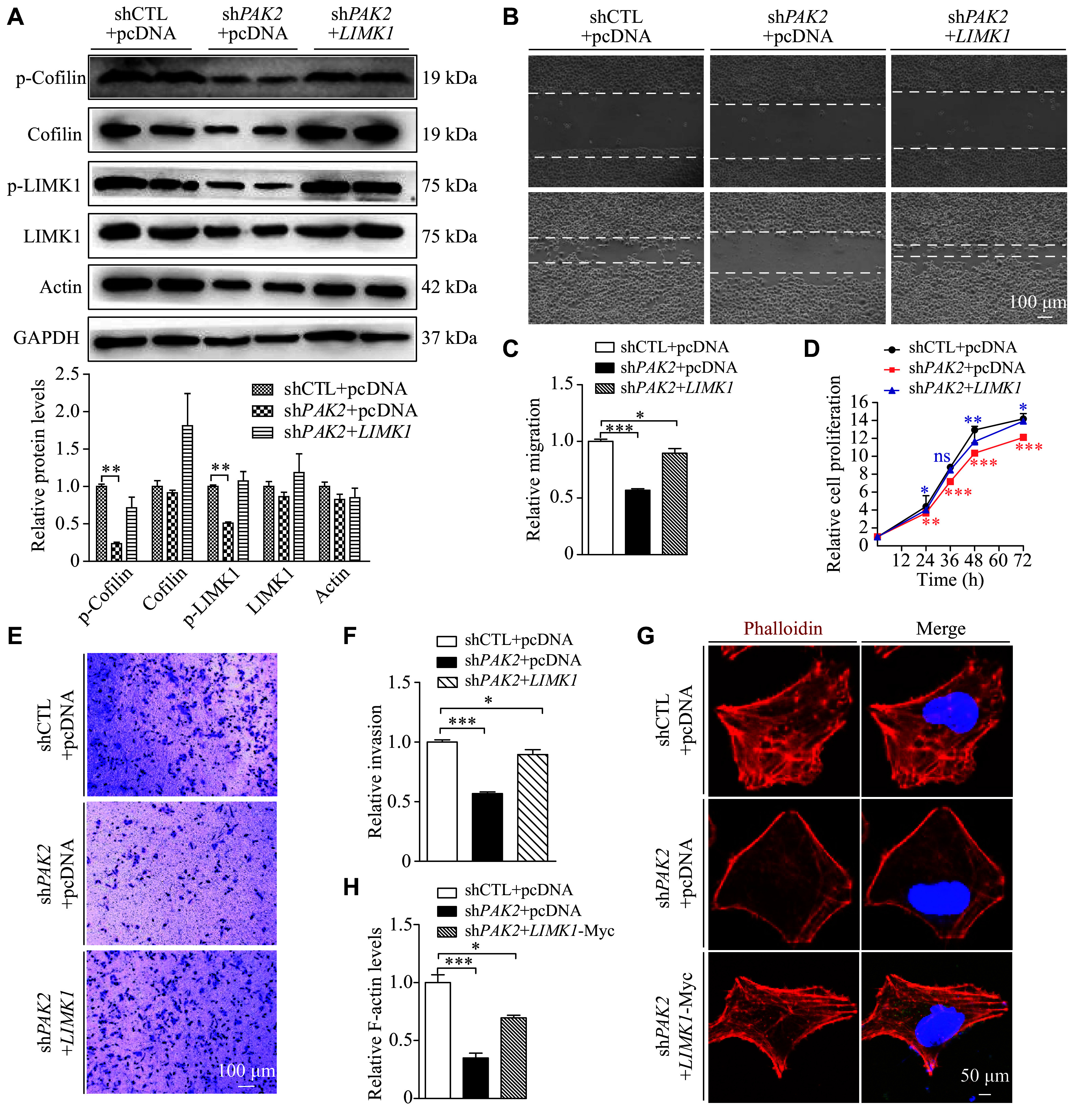
LIMK1 overexpression in *PAK2* knockdown cells partially rescued the impaired proliferative, migratory, and invasive capacities of the cells. A: The sh*PAK2* cells were transfected with pCDNA-*LIMK1*-Myc plasmids, and the expression levels of p-cofilin, cofilin, p-LIMK1, LIMK1, and actin were confirmed through Western blotting. B and C: Wound healing assays were carried out to investigate the effects of LIMK1 overexpression on migratory ability of sh*PAK2* H226 cells. The areas covered by cells and the blank spaces are separated by white dashed lines. Scale bar, 100 μm. D: CCK8 assays were performed to assess the effect of LIMK1 overexpression on the proliferation of sh*PAK2* cells. E and F: Matrigel invasion assays were conducted to explore the influence of LIMK1 overexpression on invasive ability of sh*PAK2* H226 cells. Scale bar, 100 μm. G and H: Immunofluorescence experiments were conducted to show the co-staining of F-actin and LIMK1-Myc with Myc-Tag antibody, respectively. DAPI (blue), F-actin (red), LIMK1-Myc (green), and merged images are shown. Scale bar, 50 μm. Relative F-actin levels in H226 cells (H). Data are presented as mean ± standard deviation. Statistical analyses were performed by two-tailed unpaired Student's *t*-test for two-group comparisons. ^*^P < 0.05, ^**^P < 0.01, and ^***^P < 0.001.

PAK2, as the downstream target of RAC1 and CDC42, is activated and regulates actin dynamics through the LIMK1/cofilin signaling pathway. LIMK1 phosphorylates cofilin at Ser-3, inactivating cofilin's binding to F-actin and its depolymerization activities. To verify the involvement of PAK2 in the LUSC progression through the LIMK1/cofilin signaling pathway, we transfected pCDNA-*LIMK1* plasmids into sh*PAK2* cells to overexpress LIMK1. The overexpression of LIMK1 was detected by Western blotting (***Fig. 6A***). After overexpressing LIMK1 in sh*PAK2* cells, the decreased levels of p-LIMK1, cofilin, and p-cofilin mediated by sh*PAK2* were restored, whereas the actin levels were not significantly changed (***Fig. 6A***).

LIMK1 overexpression also significantly restored cell migration, proliferation, and invasion in PAK2-deficient cells (***Fig. 6B***–***6F***). The pCDNA-*LIMK1*-Myc plasmid was then used to detect the LIMK1 through an immunostaining assay. Specifically, Myc-Tag antibodies were used to perform double immunostaining for phalloidin and Myc-LIMK1. We observed that the decreased and disrupted distribution of F-actin in sh*PAK2* cells was significantly restored upon LIMK1 overexpression (***Fig. 6G*** and ***6H***). In summary, the ability of PAK2 overexpression to partially restore cell proliferation, migration, and invasion by regulating the LIMK1/cofilin signaling pathway further underscores its significance in LUSC cells.

## Discussion

In the present study, we comprehensively analyzed the functions and mechanisms of PAK2 in LUSC progression and discovered that upregulated PAK2 expression levels in human LUSC tissues were associated with reduced survival in LUSC patients. Mechanistically, PAK2 may play an oncogenic role in LUSC progression through the LIMK1/cofilin signaling pathway. However, additional studies are needed to assess the potential of PAK2 as a therapeutic target and molecular diagnostic marker for LUSC.

Cell cycle progression depends on the coordination of various regulatory factors, and most human malignant tumors involve mutations in one or more cell cycle regulators^[24]^. Therefore, targeting cell cycle regulators may effectively inhibit tumor cell growth. The present study found that *PAK2* knockdown reduced cell proliferative activity both *in vitro* and *in vivo*. Subsequently, qRT-PCR analysis showed that *PAK2* deficiency downregulated the expression of cell cycle regulators, such as cyclin A2, cyclin D1, and cyclin E1, while upregulating the expression of cell cycle inhibitors, such as *P21* and *P27* in LUSC cells. These findings suggest that silencing *PAK2* interrupts cell cycle progression. Therefore, we hypothesize that PAK2 may indirectly modulate cell cycle regulators. However, further investigation is needed to elucidate the precise mechanism by which PAK2 regulates cell cycle processes and cell proliferation.

Studies have demonstrated that migration and invasion are critical factors affecting distant tumor metastasis and that actin cytoskeleton reorganization is essential for tumor cell migration and invasion^[25]^. Additionally, the *LIMK* gene plays an integral role in regulating actin polymerization and depolymerization. For instance, LIMK1 has been shown to promote tumor cell migration and invasion by regulating ADF/cofilin-mediated actin dynamics^[26–27]^. In the present study, we demonstrated that *PAK2* knockdown inhibited the phosphorylation of LIMK1 and cofilin, thereby decreasing F-actin levels and impairing cell morphology. Moreover, LIMK1 overexpression partially restored cell proliferation, migration, and invasion in sh*PAK2* cells. These results indicate that PAK2 may contribute to the LUSC progression through the LIMK1/cofilin-mediated actin dynamics.

Studies have reported that the p-cofilin to cofilin ratio affects actin polymerization and depolymerization^[28]^. In the present study, we found that the expression levels of p-cofilin were significantly altered between the sh*PAK2* and sh*PAK2*+LIMK1 groups, with total cofilin levels following a similar trend. Compared with the sh*PAK2* group, the p-cofilin/cofilin ratio in the sh*PAK2*+*LIMK1* group showed a mean increase of 63% (from 0.67 to 1.09), suggesting that the increased cofilin levels may serve as a negative feedback mechanism to maintain actin dynamics in cells.

Our findings suggest that cofilin may need to reach a specific phosphorylation threshold to promote migration, because the decreased cofilin phosphorylation impaired cell migration in *PAK2* knockdown cells. This observation is consistent with previous findings that blocking or over-activating RAC1 significantly reduced or increased cofilin phosphorylation, respectively, and both conditions abolished border cell migration^[29]^. These results suggest that the dynamic phosphorylation and dephosphorylation cycles at Ser-3 are indispensable for cofilin to regulate actin dynamics and regulate cell migration effectively.

On the other hand, the GTPase-activated PAKs exert their effects through kinase activities and by mediating downstream signaling events. RHO, RAC, and CDC42 are activated by various trans-membrane receptors, transmitting signals to the downstream effectors, such as ROCK1, PAK1, and PAK2^[30]^. The RHO GTPase family is primarily associated with actin cytoskeleton reorganization and motility^[31]^. However, the specific function of GTPase upstream of PAK2 remains elusive and requires further investigation.

Studies have reported that PAK2 is involved in glucose uptake in neurons and that cancer cells rapidly consume glucose to meet high energy demands^[10]^. Glucose transporter 1 (GLUT1) is the most widely distributed glucose transporter and plays a key role in fundamental glucose uptake^[32]^. At the molecular level, excessive GLUT1 activation directly enhances glucose accumulation in cancer cells and promotes their excessive growth^[33]^. Moreover, GLUT1 has been shown to promote lung cancer cell proliferation, invasion, and migration. High GLUT1 levels in LUAD are significantly associated with poor survival in patients and are often accompanied by brain and lymph node metastases^[34]^. To investigate whether PAK2 regulates glucose uptake, we examined *GLUT1* expression in LUSC cells with stable transfection of sh*PAK2* or shCTL. However, we found no significant differences in the expression levels of GLUT1 between the sh*PAK2* and shCTL cells (data not shown). Therefore, further studies should explore additional biochemical indicators, such as glucose uptake, lactate production, and ATP production, to further elucidate the role of PAK2 in cancer metabolism.

In summary, the present study demonstrated that PAK2 promoted LUSC cell proliferation, migration, and invasion, resulting in a poor prognosis for LUSC patients. Furthermore, our findings provided some evidence to support the involvement of the PAK2/LIMK1/cofilin signaling pathway in LUSC progression, suggesting potential therapeutic targets for clinical LUSC treatment.

## SUPPLEMENTARY DATA

Supplementary data to this article can be found online.
