# Supplementary material for: PAK2 promotes proliferation, migration, and invasion of lung squamous cell carcinoma through the LIMK1/cofilin signaling pathway
Source: J Biomed Res. 2024 Jun 3;39(2):184–97. doi: 10.7555/JBR.37.20230317 (PMC11982680; doi:10.7555/JBR.37.20230317)
Supplement: Supplementary file 1 — Supplementary data to this article can be found online. [file jbr-39-2-184-S1.pdf]

Available online at www.jbr-pub.org.cn

Open Access at PubMed Central

Journal of Biomedical Research, 2025 39(2): 1–6

Supplementary Data

JBR

PAK2 promotes proliferation, migration, and invasion of lung squamous cell carcinoma through the LIMK1/cofilin signaling pathway

Congcong Wang[1,△], Junyan Wang[1,△], Ruifeng Xu[1,△], Qiushuang Li[1], Xia Huang[1], Chenxi Zhang[2,✉], Baiyin Yuan[1,✉]

[1]College of Life and Health Sciences, Wuhan University of Science and Technology, Wuhan, Hubei 430081, China; [2]Central Laboratory, Nanjing Chest Hospital, Affiliated Nanjing Brain Hospital of Nanjing Medical University, Nanjing, Jiangsu 210029, China.

Supplementary Table 1 **Characteristics of non-small cell lung cancer patients who participated in quantitative reverse transcription-PCR (qRT-PCR) and copy number detection**

Patient No.	Sex	Age (years)	Type of pathology	Smoking history	Stage	Sample use
1	Male	77	LUAD	Yes	Ⅲa	qRT-PCR
2	Male	64	LUAD	Yes	Ⅲa	qRT-PCR
3	Male	70	LUAD	Yes	Ⅲa	qRT-PCR
4	Male	67	LUAD	No	Ⅰb	qRT-PCR
5	Female	67	LUAD	No	Ⅰb	qRT-PCR
6	Male	66	LUAD	Yes	Ⅲa	qRT-PCR
7	Male	51	LUAD	No	Ⅲa	qRT-PCR
8	Female	66	LUAD	No	Ⅱa	qRT-PCR
9	Male	73	LUAD	No	Ⅲa	qRT-PCR
10	Male	61	LUAD	Yes	Ⅰa	qRT-PCR
11	Male	67	LUAD		Ⅱa	qRT-PCR
12	Male	62	LUAD	Yes	Ⅲa	qRT-PCR
13	Female	53	LUAD	No	Ⅰb	qRT-PCR
14	Female	67	LUAD	No	Ⅰb	qRT-PCR
15	Male	62	LUAD	Yes	Ⅱb	qRT-PCR
16	Male	50	LUAD	No	Ⅳ	qRT-PCR

△These authors contributed equally to this work.

✉Corresponding authors: Chenxi Zhang, Central Laboratory, Nanjing Chest Hospital, Affiliated Nanjing Brain Hospital of Nanjing Medical University, 215 Guangzhou Road, Gulou, Nanjing, Jiangsu 210029, China. E-mail: chenxi4262@njmu.edu.cn; Baiyin Yuan, College of Life and Health Sciences, Wuhan University of Science and Technology, 947 Heping Avenue, Qingshan District, Wuhan, Hubei 430081, China. E-mail: yuanby@wust.edu.cn.

Received: 26 December 2023; Revised: 28 April 2024; Accepted: 27 May 2024; Published online: 03 June 2024

CLC number: R734.2, Document code: A

The authors reported no conflict of interests.

This is an open access article under the Creative Commons Attribution (CC BY 4.0) license, which permits others to distribute, remix, adapt and build upon this work, for commercial use, provided the original work is properly cited.

© 2025 by Journal of Biomedical Research.

https://doi.org/10.7555/JBR.37.20230317

Table 1 **Characteristics of non-small cell lung cancer patients who participated in quantitative reverse transcription-PCR (qRT-PCR) and copy number detection** (Continued)

Patient No.	Sex	Age (years)	Type of pathology	Smoking history	Stage	Sample use
17	Female	50	LUAD	No	Ⅰa	qRT-PCR
18	Male	65	LUAD	No	Ⅳ	qRT-PCR
19	Female	68	LUAD	No	Ⅰa	qRT-PCR
20	Female	65	LUAD	No	Ⅰa	qRT-PCR
21	Female	66	LUAD	No	Ⅱb	qRT-PCR
22	Male	70	LUAD	Yes	Ⅲa	qRT-PCR
23	Female	69	LUAD	No	Ⅲa	qRT-PCR
24	Male	60	LUAD	Yes	Ⅳ	qRT-PCR
25	Male	60	LUSC	Yes	Ⅰb	qRT-PCR and copy number
26	Male	63	LUSC	Yes	Ⅲb	qRT-PCR and copy number
27	Male	78	LUSC	Yes		qRT-PCR and copy number
28	Male	71	LUSC	Yes	Ⅰb	qRT-PCR and copy number
29	Male	62	LUSC	Yes	Ⅰb	qRT-PCR and copy number
30	Male	52	LUSC	Yes	Ⅲa	qRT-PCR and copy number
31	Male	65	LUSC	Yes	Ⅰb	qRT-PCR and copy number
32	Male	59	LUSC	Yes	Ⅰb	qRT-PCR and copy number
33	Male	63	LUSC	–	Ⅲb	qRT-PCR and copy number
34	Male	63	LUSC	Yes	Ⅲa	qRT-PCR and copy number
35	Male	70	LUSC	Yes	Ⅱa	qRT-PCR and copy number
36	Male	69	LUSC	Yes	Ⅱb	qRT-PCR and copy number
37	Male	72	LUSC	Yes	Ⅰb	qRT-PCR and copy number
38	Male	57	LUSC	Yes	Ⅲa	qRT-PCR and copy number
39	Male	69	LUSC	Yes	Ⅲa	qRT-PCR and copy number
40	Male	68	LUSC	Yes	Ⅲa	qRT-PCR and copy number
41	Male	70	LUSC	Yes	Ⅱb	qRT-PCR and copy number
42	Male	69	LUSC	Yes	Ⅲa	qRT-PCR and copy number
43	Male	71	LUSC	Yes	Ⅰb	Copy number
44	Male	75	LUSC	No	Ⅲa	Copy number
45	Male	60	LUSC	No	Ⅰa	Copy number
46	Male	66	LUSC	Yes	Ⅲb	Copy number
47	Male	76	LUSC	Yes	Ⅰb	Copy number
48	Male	69	LUSC	Yes	Ⅰa	Copy number
49	Male	69	LUSC	No	Ⅱa	Copy number
50	Male	58	LUSC	Yes	Ⅲa	Copy number
51	Male	67	LUSC	Yes	Ⅱb	Copy number
52	Male	68	LUSC	Yes	Ⅱa	Copy number
53	Male	72	LUSC	Yes	Ⅰa	Copy number
54	Male	52	LUSC	Yes	Ⅳ	Copy number

Abbreviations: LUAD, lung adenocarcinoma; LUSC, lung squamous cell carcinoma. En dash indicates the missing data because of incomplete provision of patient information.

Supplementary Table 2 **Characteristics of non-small cell lung cancer patients who participated in immunohistochemical microarrays and survival curves**

Patient No.	Age (years)	Sex	Stage	Scores	Survival (months)	States of survival
1	61	Male	T2a	0	81	Death
2	72	Male	T4	2	91	Alive
3	62	Male	T2b	0	89	Alive
4	57	Male	T1b	0	87	Alive
5	61	Male	T1a	4	86	Alive
6	64	Male	T2a	9	86	Alive
7	78	Male	T1a	0	70	Death
8	68	Male	T4	1	84	Alive
9	56	Male	T1b	0	84	Alive
10	63	Male	T2a	0	84	Alive
11	53	Male	T2b	4	80	Alive
12	57	Male	T3-T4	4	80	Alive
13	57	Male	T2b	1	80	Alive
14	65	Male		0	78	Alive
15	58	Male	T2b	3	59	Death
16	62	Male	T2a	2	74	Alive
17	67	Male	T3	0	69	Alive
18	72	Male	T3	9	34	Death
19	60	Male	T2b	2	10	Death
20	73	Male	T2b	2	4	Death
21	68	Male	T2a	9	68	Alive
22	52	Male	T2b	9	29	Death
23	63	Male	–	9	67	Alive
24	75	Male	–	2	66	Alive
25	64	Male	–	1	66	Alive
26	70	Male	T2a	6	64	Alive
27	59	Male	T2a	1	64	Alive
28	58	Male	T2a	9	64	Alive
29	48	Male	T2a	6	63	Alive
30	52	Male	T2a	6	63	Alive
31	76	Male	T3	9	63	Alive
32	78	Male	T2b	9	29	Death
33	55	Male	T3	6	62	Alive
34	56	Male	T2a	4	13	Death
35	68	Male	T2a	2	61	Alive
36	75	Male	T2a	6	2	Death
37	64	Male	T3	9	10	Death
38	72	Male	T2b	9	45	Death
39	67	Male	T2a	6	41	Death
40	72	Male	T2a	6	50	Death
41	52	Male	T2a	6	59	Alive
42	70	Male	T1a	2	16	Death
43	63	Male	T2b	6	57	Alive
44	70	Male	T2b	9	16	Death

Table 2 **Characteristics of non-small cell lung cancer patients who participated in immunohistochemical microarrays and survival curves** (Continued)

Patient No.	Age (years)	Sex	Stage	Scores	Survival (months)	States of survival
45	73	Male	–	9	1	Death
46	55	Male	T1b	4	38	Death
47	59	Male	T3	9	57	Alive
48	53	Male	T2b	2	56	Alive
49	72	Male	T1b	3	8	Death
50	63	Male	T3	9	54	Death
51	70	Male	T3	9	16	Death
52	60	Male	T2a	4	54	Alive
53	57	Male	T3	6	53	Death
54	57	Male	T2a	1	53	Alive
55	75	Male	T2b	6	22	Death
56	74	Male	T1b	2	46	Death
57	76	Male	T2a	6	0	Death
58	55	Male	T2a	2	4	Death
59	–	Male	T3	2	52	Alive
60	–	Male	T2a	1	52	Alive

En dash indicates the missing information.

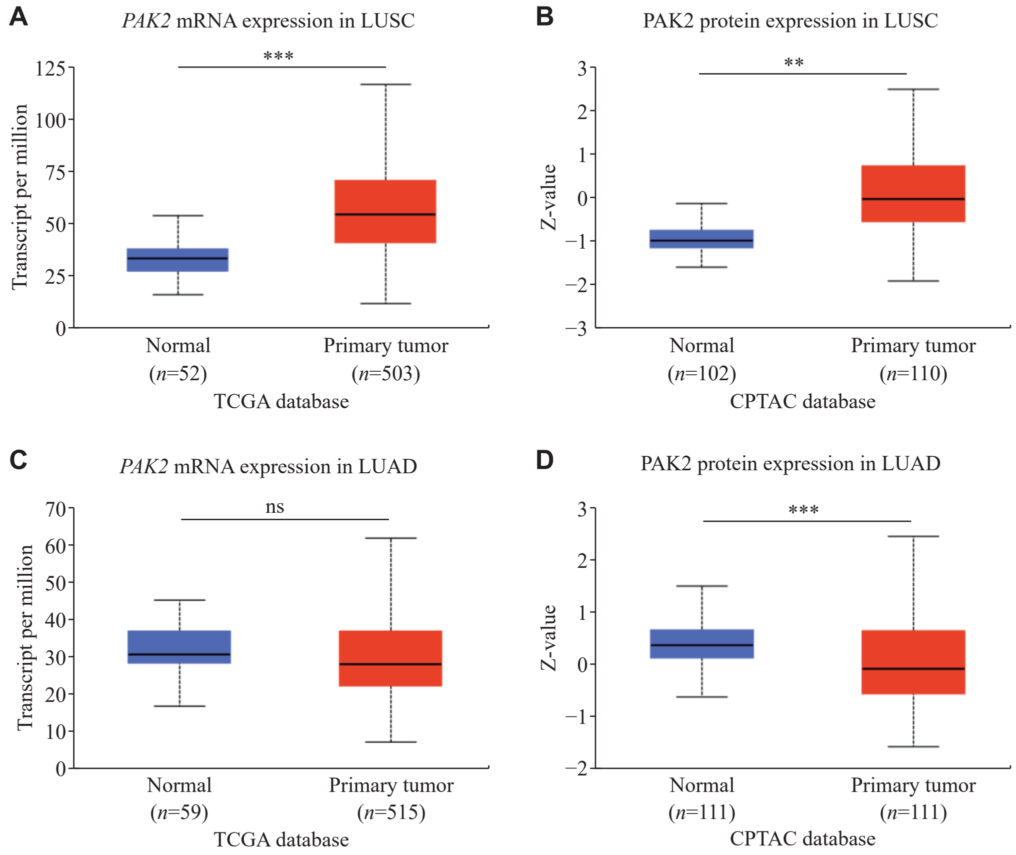

Supplementary Fig. 1 **The elevated PAK2 expression in LUSC tissues.** A: The mRNA expression of *PAK2* in LUSC from the TCGA database. Red represents primary tumor samples (n = 52), and blue represents normal samples (n = 503). B: The protein expression of PAK2 in LUSC from the CPTAC database. The primary tumor samples (n = 110) and normal samples (n = 102) were reported as red and blue, respectively. C: The mRNA expression of *PAK2* in LUAD from the TCGA database. Red represents primary tumor samples (n = 59), and blue represents normal samples (n = 515). D: The protein expression of PAK2 in LUAD from the CPTAC database. Red, primary tumor samples (n = 111). Blue, normal samples (n = 111). $^{**}P$ < 0.01 and $^{***}P$ < 0.001. Abbreviations: TCGA, The Cancer Genome Atlas. LUSC, lung squamous cell carcinoma. LUAD, lung adenocarcinoma. CPTAC, Clinical Proteomic Tumor Analysis Consortium; ns, not significant.

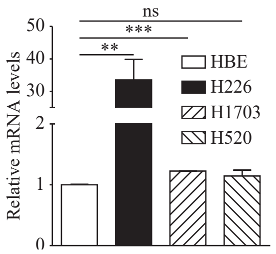

Supplementary Fig. 2 **The qRT-PCR analysis of *PAK2* expression levels in LUSC cells.** The qRT-PCR analysis of *PAK2* expression levels in HBE, H226, H1703, and H520 cells. Data of relative mRNA levels (y-axis) from three independent experiments were normalized to *GAPDH* and were presented as mean ± standard deviation. $^{**}P < 0.01$ and $^{***}P < 0.001$ by two-tailed unpaired Student's *t*-test. Abbreviation: ns, not significant.

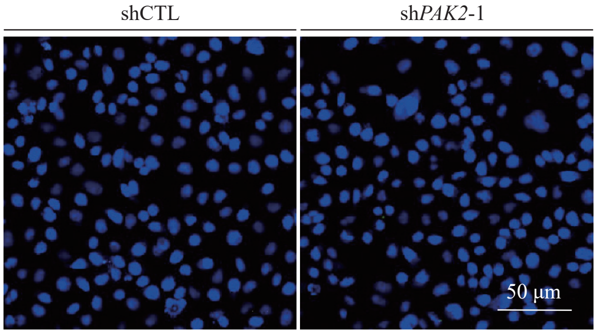

Supplementary Fig. 3 **TUNEL staining of H226 cells.** TUNEL staining was used to detect cell apoptosis levels in control and *PAK2* knockdown H226 cells. The merged pictures with DAPI (blue) and apoptosis marker (green) are shown. Scale bar, 50 μm.

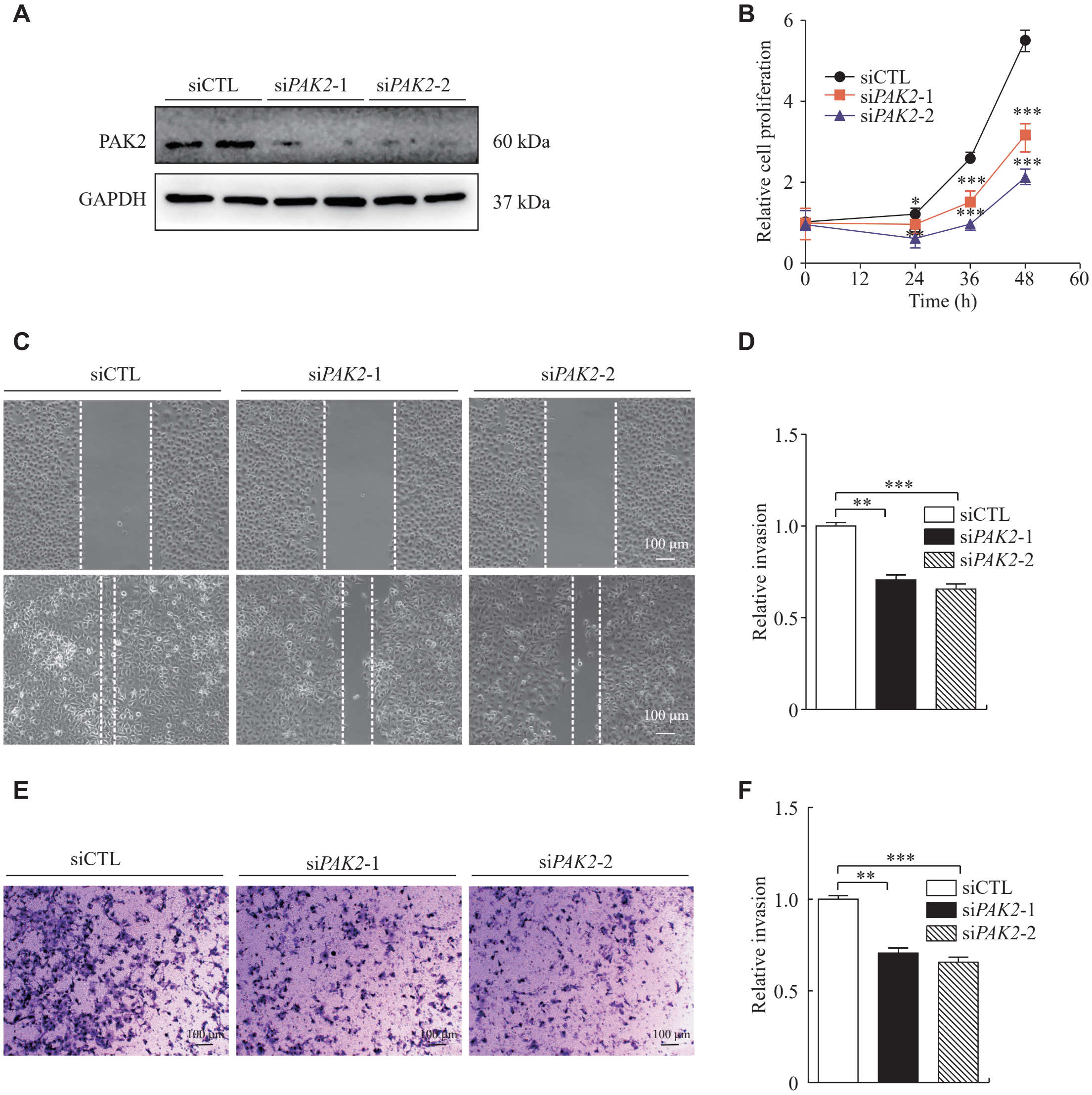

Supplementary Fig. 4 **PAK2 silencing suppressed the proliferation, migration, and invasion of H520 cells.** A: The relative PAK2 expression levels in H520 cells of the si*PAK2*-1 group and si*PAK2*-2 group were determined by Western blotting. B: CCK8 assay was used to test the effect of *PAK2* knockdown on cell proliferation rate of H520 cells. C and D: The effect of *PAK2* knockdown in H520 cells on cell migration was analyzed by wound healing (C) and quantification analysis (D). Scale bar, 100 μm. E and F: Matrigel invasion assay (E) and quantification analysis (F) were used to show the change of *PAK2* silencing on cell invasive ability of H520 cells. Scale bar, 100 μm. Data are presented as mean ± standard deviation. Statistical analyses were performed by two-tailed unpaired *t*-test for two-group comparisons. $^{**}P < 0.01$ and $^{***}P < 0.001$.

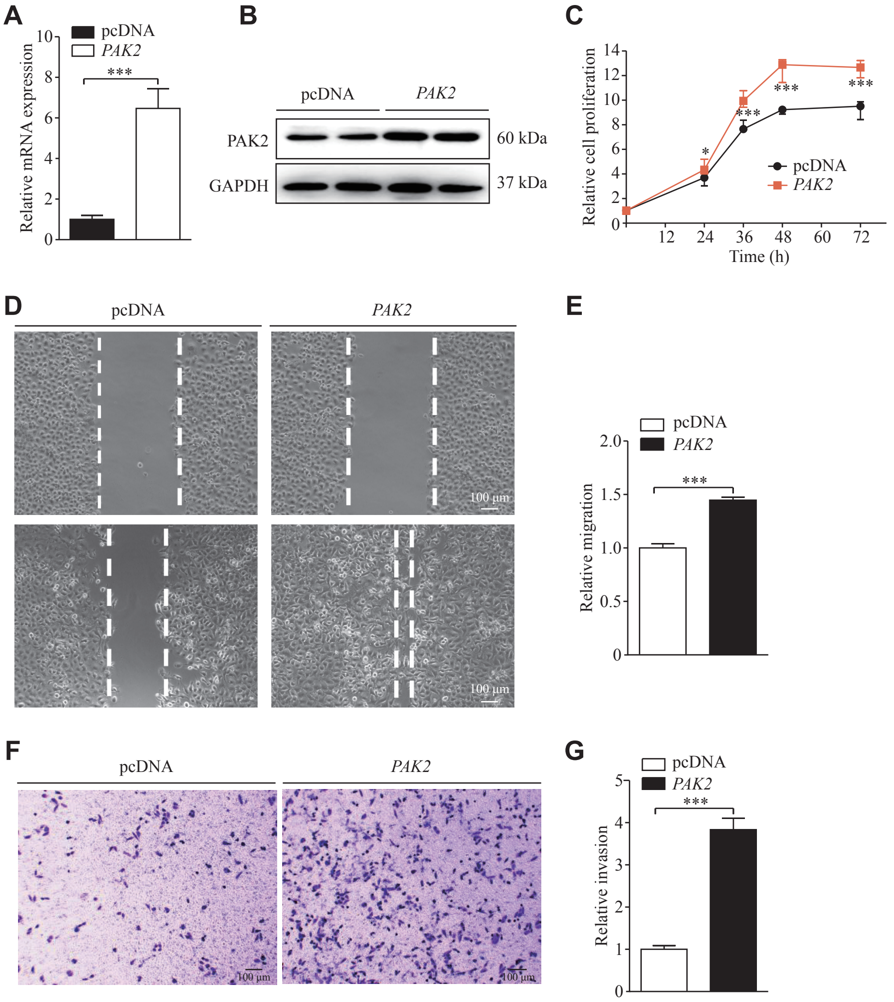

Supplementary Fig. 5 **Overexpression of PAK2 promoted the proliferation, migration, and invasion of LUSC cells.** A and B: The efficiency of PAK2 overexpression in H226 cells was determined by qPCR (A) and Western blotting (B). C: CCK8 assay was performed to test the effect of PAK2 overexpression on the cell proliferation in H226 cells. D and E: Wound healing assay (D) and quantification analysis (E) of PAK2 overexpression and pCDNA group H226 cells. Scale bar, 100 μm. F and G: Transwell assays (F) and quantification analysis (G) of PAK2 overexpression on cell invasive ability of H226 cells. Scale bar, 100 μm. Data are presented as mean ± standard deviation. Statistical analyses were performed by two-tailed unpaired Student's *t*-test for two-group comparisons. $^{*}P < 0.05$ and $^{***}P < 0.001$.

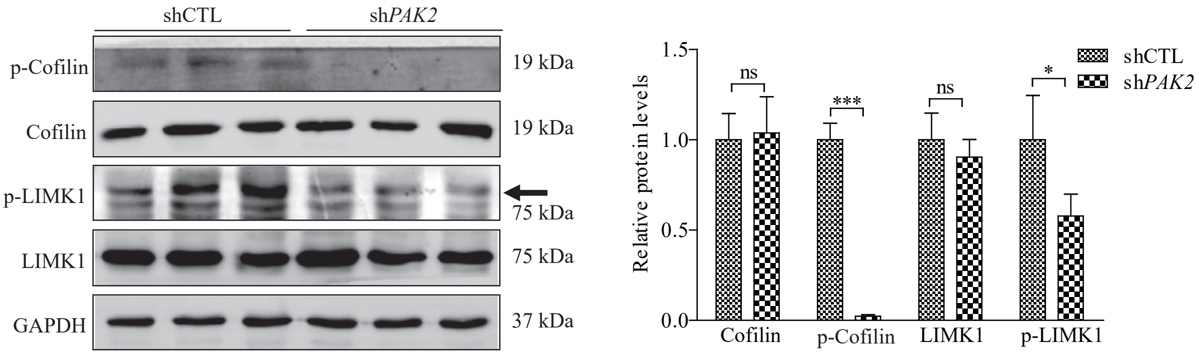

Supplementary Fig. 6 ***PAK2*** **knockdown led to a decrease in the phosphorylation of cofilin and LIMK1 in xenograft tumors.** Western blotting analysis was performed to assess the levels of p-cofilin, cofilin, p-LIMK1, and LIMK1 in xenograft tumors with CTL and *PAK2* knockdown mice. $^{*}P < 0.05$ and $^{***}P < 0.001$ by two-tailed unpaired Student's *t*-test. Abbreviation: ns, not significant.
